# Intelligent robot-assisted fracture reduction for pelvic fractures: a clinical study

**DOI:** 10.3389/fmed.2026.1744048

**Published:** 2026-02-18

**Authors:** Xinyu Fan, Jianlin He, Baochuang Qi, Hu Zhang, Gang Li, Xingqiang Liu, Wei Yu, Nuocheng Yang, Yin Yang, Yongqing Xu

**Affiliations:** 1 920th Hospital of Joint Logistics Support Force, Kunming, Yunnan, China; 2 Orthopedic Trauma Center, Yunnan Provincial Hospital of Traditional Chinese Medicine/The First Affiliated Hospital of Yunnan University of Chinese Medicine, Kunming, Yunnan, China; 3 Department of Orthopedics, Hospital of Guizhou Panjiang Coal and Electricity Group Co., Ltd., Guizhou, China; 4 School of Clinical Medicine, Dali University, Dali, Yunnan, China; 5 Department of Orthopaedics, Dali Bai Autonomous Prefecture People’s Hospital, Dali, Yunnan, China

**Keywords:** intelligence, minimally invasive, pelvic fractures, reduction, robot

## Abstract

**Objective:**

The objective of the study was to evaluate the safety, feasibility, and radiographic outcomes of an intelligent robot-assisted fracture reduction (RAFR) system in the minimally invasive treatment of fresh, unstable pelvic ring injuries.

**Methods:**

In this single-center retrospective case series, 32 consecutive patients with unstable pelvic ring injuries (Tile type B or C) treated between August 2024 and April 2025 underwent minimally invasive closed reduction and internal fixation using the RAFR system. The system combines preoperative computed tomography (CT)-based three-dimensional reduction planning, intraoperative cone-beam CT (CBCT) registration, an optical tracking system, a table-mounted passive holding arm, and a robotic arm with dual force–position monitoring. Operative time, intraoperative blood loss, and fluoroscopic exposures were recorded. Postoperative CT was used to measure residual displacement, which was graded according to Matta’s criteria, and the excellent-to-good rate was calculated. Functional outcomes were assessed using the Majeed score at the final follow-up.

**Results:**

All 32 procedures were completed using a closed, minimally invasive approach without conversion to open reduction. The median (IQR) operative time was 270 (225–311) min, blood loss was 150 (100–300) mL, and fluoroscopic exposures were 35 (30–45). The median residual displacement on CT was 4.0 (3.0–8.0) mm. According to Matta’s criteria, 17 patients (53.1%) had excellent, 12 (37.5%) had good, and 3 (9.4%) had fair reductions, yielding an excellent-to-good rate of 90.6%. Two patients were lost to follow-up; among the remaining 30 patients, no major complications such as deep infection, implant failure, or iatrogenic neurovascular injury were observed, and the mean Majeed score was 76.7 ± 12.0.

**Conclusion:**

The RAFR system enabled closed reduction and percutaneous fixation of a heterogeneous cohort of unstable pelvic ring fractures with high rates of satisfactory reduction and favorable short-term functional recovery. These preliminary findings support the clinical feasibility and safety of robot-assisted closed reduction for unstable pelvic fractures and provide a basis for future comparative and multicenter studies.

## 1 Introduction

High-energy pelvic ring injuries are among the most challenging problems in orthopedic trauma. They are frequently associated with hemodynamic instability, multiple organ injuries, substantial early mortality, and a high risk of long-term disability (1–3). The primary surgical goal is to restore pelvic ring symmetry and stability to allow early mobilization and to reduce chronic pain and functional impairment (4). Traditional open reduction and internal fixation (ORIF) can achieve anatomic or near-anatomic reduction but requires extensive soft tissue dissection, is associated with considerable blood loss and wound complications, and may be poorly tolerated in polytrauma patients (1, 4).

For these reasons, less-invasive strategies—particularly closed or limited-exposure reduction combined with percutaneous fixation—have increasingly been adopted (3, 5–7). However, conventional closed reduction techniques typically rely on Schanz pins, traction tables, or reduction frames, guided only by two-dimensional (2D) fluoroscopy (5, 8–10). These approaches are technically demanding, operator-dependent, and may still fail to achieve acceptable alignment, especially in markedly displaced or vertically unstable patterns. Surgeons must interpret complex three-dimensional (3D) deformities from 2D projections and maintain reduction manually under substantial soft tissue resistance, often at the cost of prolonged fluoroscopy and physical fatigue (5, 6, 11–13).

With the development of digital orthopedics, computer navigation and surgical robots have improved the precision of percutaneous screw placement, particularly in the sacroiliac and trans-sacral corridors (5, 14–16). However, the majority of systems focus on the fixation step rather than on actively performing the reduction maneuver itself. To address this gap, our group developed an intelligent robot-assisted fracture reduction (RAFR) system that integrates CT-based 3D planning, intraoperative CBCT registration, optical navigation, a table-mounted passive holding arm, an elastic traction device, and a force-controlled robotic arm (17–23). Preclinical studies in bone models and cadaveric specimens revealed that this system can execute planned reduction trajectories with high accuracy and repeatability (17–22).

Following these experimental validations and early clinical experiences, further clinical evidence in a broader patient cohort is needed. Therefore, the objective of this study was to describe the early clinical application of the RAFR system in 32 consecutive patients with fresh, unstable pelvic ring injuries, focusing on (1) reduction quality assessed by CT-based residual displacement and Matta grading, (2) perioperative parameters including operative time, blood loss, and fluoroscopic exposure, and (3) short-term functional outcomes.

## 2 Materials and methods

### 2.1 Study design and patient selection

This was a single-center retrospective case series conducted at a tertiary trauma center. The study was approved by the institutional ethics committee, and all patients (or their legal guardians) provided informed consent for the robotic procedure and subsequent data use.

Between August 2024 and April 2025, patients with acute unstable pelvic ring injuries were screened. The inclusion criteria were: (1) pediatric and adult patients; (2) fresh, closed, unstable pelvic ring fractures classified as Tile type B or C; and (3) hemodynamic stability at the time of definitive surgery, either primarily or after initial resuscitation. The exclusion criteria were: (1) open pelvic fractures or severe soft tissue degloving; (2) prior pelvic surgery or pre-existing pelvic deformity; (3) severely osteoporotic iliac bone, judged unable to support stable holding pins; and (4) bilateral posterior ring comminution or deformity, precluding the use of the contralateral hemipelvis as a reliable template.

The RAFR system requires an intact or minimally displaced contralateral hemipelvis for “mirror-template” planning of the reduction pathway. Patients in whom such a template could not be established were excluded from robotic reduction and treated with conventional open or manual closed techniques. After applying these criteria, 32 patients were included (16 men and 16 women). The cohort comprised both Tile type B and C injuries, and the majority of patients had associated injuries, such as thoracic trauma, lumbar fractures, or urogenital injury (Table 1). The median interval from injury to surgery was 7 days, reflecting the need to stabilize concomitant injuries and coordinate logistics.

**Table 1:** Demographic and clinical variables of the patients treated for unstable pelvic fractures.

Case number	Gender	Age (years)	BMI	Injury to surgery time (days)	Tile type	Combined injury
1	Female	33	20.06	10	B3	Scapular fracture
2	Male	58	20.33	6	B3	Urethral injury, rib fracture, and lumbar vertebra fracture
3	Male	53	21.41	7	B2	Retroperitoneal hematoma, renal contusion, traumatic pancreatitis, abdominopelvic effusion, gluteal soft tissue injury, and traumatic hemorrhagic anemia
4	Male	61	23.18	12	B3	Rib fracture, hemopneumothorax, pleural effusion, thoracic vertebra fracture, liver laceration, and renal laceration
5	Male	67	21.71	4	B3	Pulmonary contusion and rib fracture
6	Female	36	19.53	5	B2	None
7	Female	6	15.65	1	B2	None
8	Female	50	33.20	16	C3	Pulmonary contusion, rib fracture, thoracic vertebra fracture, and femoral fracture
9	Female	35	21.63	5	C1	Scapular fracture, lumbar vertebra fracture, renal contusion, rib fracture, pulmonary contusion, and hepatic contusion
10	Male	34	28.73	8	B2	None
11	Male	47	27.73	12	C2	Urethral injury and status post cystostomy
12	Female	70	23.52	8	B3	None
13	Female	31	23.82	4	B3	Lumbar vertebra fracture
14	Male	52	29.37	14	B3	None
15	Female	59	27.54	7	C1	Central hip dislocation
16	Female	38	30.08	6	B2	Pulmonary contusion and rib fracture
17	Female	42	23.23	6	B2	Sacral fracture and lumbar vertebra fracture
18	Female	42	22.22	4	B2	Distal radius fracture and ulnar styloid fracture
19	Male	63	22.83	11	C2	Pulmonary contusion, rib fracture, and pleural effusion
20	Male	48	19.57	6	B2	None
21	Male	61	22.49	3	C1	Lumbar vertebra fracture, renal contusion with subcapsular hematoma, and perirenal fascial hematoma
22	Female	55	24.65	7	C1	Pulmonary contusion, rib fracture, pleural effusion, and pneumothorax
23	Male	35	22.97	3	B2	Lumbar vertebra fracture
24	Female	14	15.55	2	B1	Abdominal soft tissue contusion
25	Male	24	32.49	2	C1	Lumbar vertebra fracture and femoral head fracture with hip dislocation
26	Female	52	19.81	2	B2	Urethral injury and lumbar vertebra fracture
27	Male	21	25.71	3	C1	Intertrochanteric femoral fracture, tibiofibular fracture, tibial plateau fracture, and lumbar vertebra fracture
28	Male	70	27.16	13	B1	Distal radius and ulnar styloid fracture, hypertension, gastrointestinal bleeding, and pulmonary contusion
29	Female	23	14.87	5	B2	None
30	Male	26	17.99	8	B2	None
31	Male	37	24.16	9	C1	Lumbar vertebra fracture
32	Female	60	19.53	8	C1	Distal radius fracture, ulnar styloid fracture, lumbar vertebra fracture, fibular fracture, and bladder injury

### 2.2 Robotic system and auxiliary devices

The intraoperative robotic system (model RR01, Beijing Rossum Robot Co., Ltd., Beijing, China) enables real-time 3D navigation, preoperative planning of fracture reduction trajectories, and execution of reduction maneuvers with dual position–force monitoring. The system comprises a master console cart with an integrated optical tracking camera, a robotic arm cart carrying the 6-degree-of-freedom collaborative robotic arm, and two key auxiliary devices: a table-mounted passive holding arm for contralateral pelvic fixation and a table-mounted elastic traction device for controlled supracondylar femoral traction (19–21).

**Figure 1:**
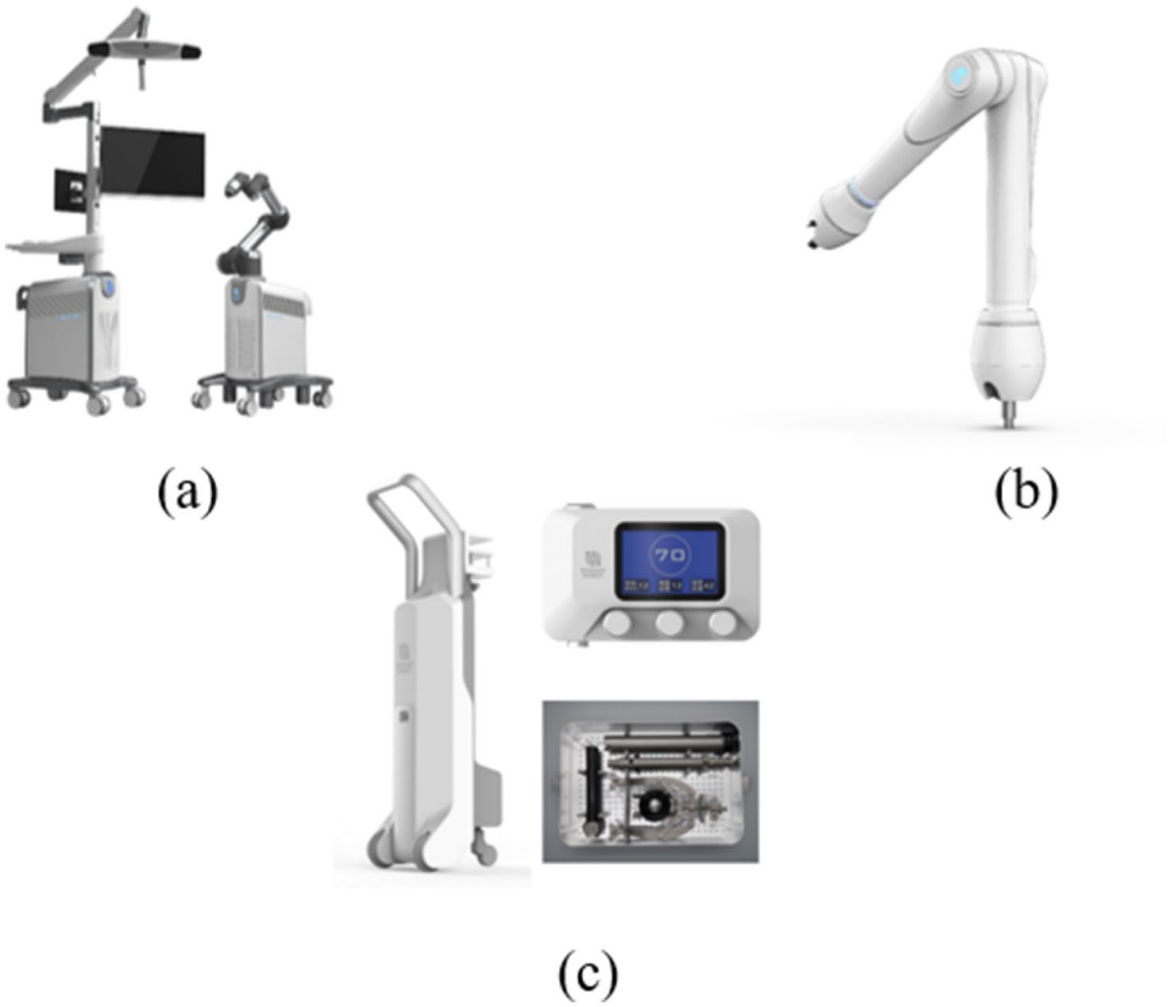
Intelligent RAFR system and auxiliary hardware. **(a)** The main robotic platform, comprising the master console cart with an integrated optical tracking system (left) and the robotic arm cart carrying the 6-degree-of-freedom collaborative arm (right). **(b)** The table-mounted passive holding arm used for rigid stabilization of the intact contralateral hemipelvis. **(c)** The table-mounted elastic traction device designed to provide continuous supracondylar femoral traction for offloading soft tissue tension.

The passive holding system incorporates two electronically controlled passive arms with multiple degrees of freedom (Pass Arm 1, Rossum Robot Co., Ltd.), which can be rigidly connected to supra-acetabular and iliac crest holding pins on the intact hemipelvis (21, 23). The elastic traction device delivers a programmable traction force of 0–30 kg through a femoral supracondylar pin, maintaining near-constant axial traction to counteract soft tissue resistance while limiting the load transmitted to the robotic arm (20, 21, 24) (Figure 1).

### 2.3 Preoperative planning

Preoperative pelvic CT data in DICOM format were imported into proprietary RROS software (Rossum Robot Co., Ltd.) for segmentation of the bony pelvis and identification of the injured and uninjured hemipelves. The software generated a mirrored 3D model of the intact hemipelvis and automatically computed a target reduction position and trajectory for the injured side based on pelvic symmetry and pre-defined anatomical constraints (17, 22). The operating surgeon reviewed and, if necessary, refined the proposed reduction pathway to avoid potential collisions and to accommodate fracture-specific features. Once approved, the plan was stored as an .rrs file and transferred to the robotic console.

### 2.4 Surgical technique

All patients were positioned supine on a radiolucent orthopedic table with the sacrum slightly elevated. After standard preparation and draping, trackers were fixed to both anterior superior iliac spines. Anteroposterior (AP) and lateral fluoroscopic images were acquired to define the CBCT scanning range, after which an intraoperative CBCT scan of the pelvis was performed. The intraoperative CBCT dataset was automatically registered to the preoperative CT-based 3D model. The optical tracking system then continuously monitored pelvic and tracker positions to provide real-time navigation.

Under navigational guidance, two Schanz pins were inserted into the intact hemipelvis (LC-2 corridor and iliac crest) and connected to the passive holding arms, providing rigid contralateral fixation outside the future implant corridors (21, 23). On the injured side, two corresponding pins and one additional supra-acetabular pin were placed and attached to the robotic arm. For vertically displaced patterns, supracondylar traction pins were inserted into the femur and connected to the elastic traction device, which was set to deliver a predefined traction force based on fracture configuration and patient characteristics (20, 21).

The robotic arm then executed the preplanned reduction pathway in a stepwise manner. The console displayed real-time fragment position and multidirectional forces. If resistance in any axis exceeded the safety threshold, the system automatically paused and switched to an assistive mode, prompting the surgeon to release interlocking fragments or adjust pin configuration. Once forces returned to within the safe range, autonomous reduction resumed until the target position was achieved, at which point both the robotic and passive arms were locked to maintain reduction.

**Figure 2:**
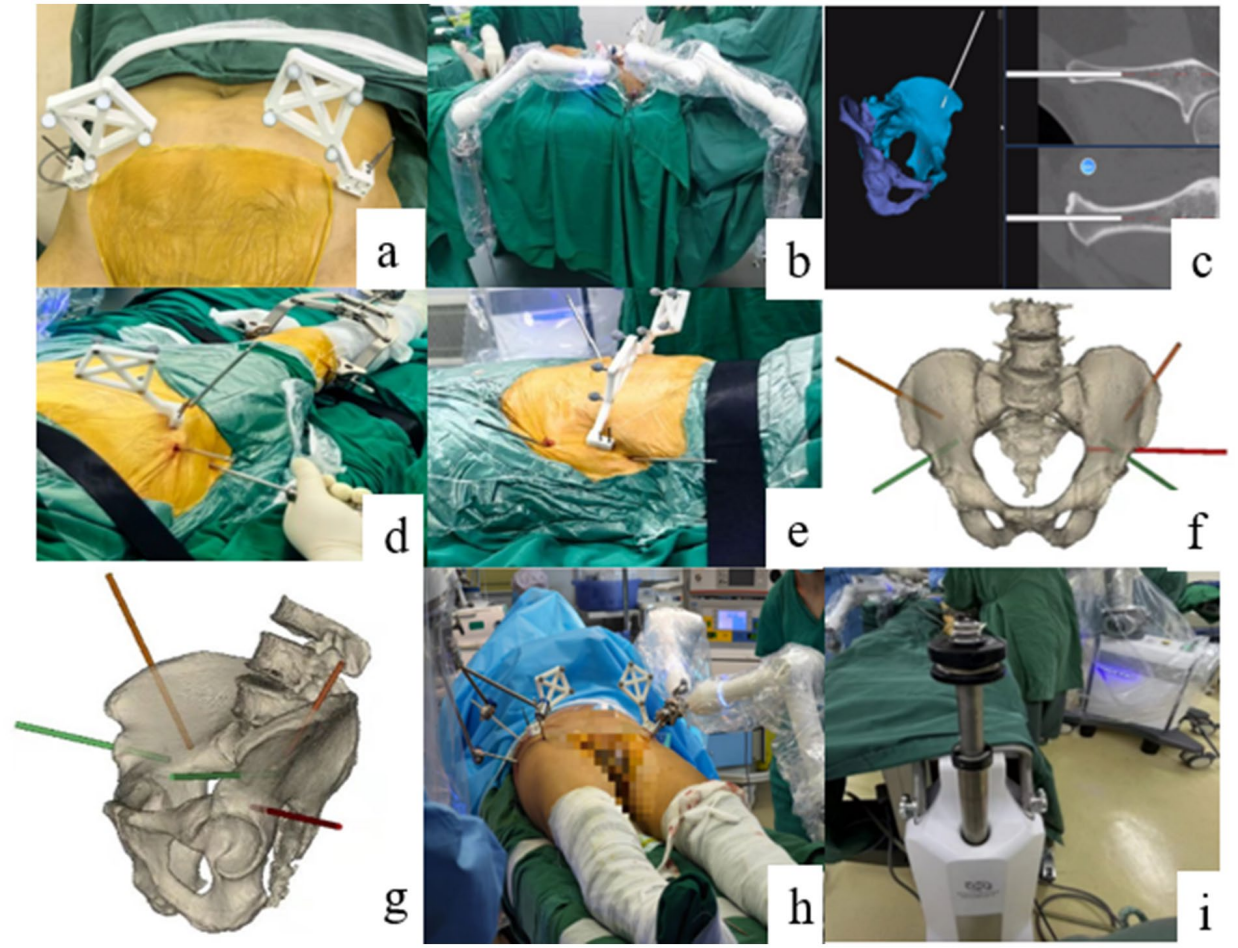
Intraoperative surgical workflow and robotic-patient linkage. **(a)** Fixation of the optical trackers on the bilateral anterior superior iliac spines (ASIS). **(b)** Sterile draping of the passive holding arms prior to mechanical connection. **(c)** Real-time navigated insertion of the pelvic holding pins. **(d–g)** Schematic diagrams illustrating the standardized placement of holding pins for the intact (contralateral) and injured (ipsilateral) hemipelves. **(h)** Final intraoperative configuration showing the rigid linkage between the pelvic pins, the robotic arm, and the passive arms. **(i)** Integration of the femoral supracondylar traction pin with the table-mounted elastic traction device.

Following the reduction, pelvic AP, inlet, and outlet fluoroscopic views were obtained to confirm alignment. Percutaneous iliosacral, trans-sacral, LC-2, and pubic screws were then inserted under either navigation or fluoroscopy according to fracture morphology. When indicated, INFIX or external fixation constructs were used for anterior ring stabilization. Final fluoroscopic images confirmed reduction and implant position before wound closure (Figure 2).

### 2.5 Postoperative management and follow-up

Postoperative care followed institutional pelvic fracture protocols. Prophylactic antibiotics were administered for up to 24 h, tailored to soft tissue conditions (2). Thromboprophylaxis consisted of low-molecular-weight heparin combined with intermittent pneumatic compression and elastic stockings for approximately 4 weeks or until full ambulation (2, 8, 9). Early active and passive exercises of the lower limbs were encouraged. For unilateral injuries, patients were allowed to bear weight on the uninjured side immediately, while the injured side was kept non-weight-bearing for approximately 3 months. Bilateral injuries required non-weight-bearing on both sides for a similar period. For LC-1-type patterns, partial weight-bearing on the injured side was allowed earlier with close radiographic surveillance (8, 9).

Follow-up was scheduled at 4, 8, and 12 weeks after surgery. At each visit, pelvic radiographs were obtained to assess fracture healing and implant integrity, and complications were recorded. At the final visit, the Majeed score was used to assess pelvic function.

### 2.6 Outcome measures

Radiographic reduction quality was primarily assessed on postoperative 3D CT reconstructions and multiplanar reformats (axial, coronal, and sagittal). Residual displacement was defined as the maximum gap or step-off at the fracture site on the slice demonstrating the greatest deformity. Two senior observers (radiologists or orthopedic surgeons) not involved in the index surgery independently measured residual displacement; the mean of the two measurements was used for analysis. Based on these measurements, the reduction quality was graded according to Matta’s criteria as excellent (≤ 4 mm), good (5–10 mm), fair (10–20 mm), or poor (> 20 mm), and the excellent-to-good rate was calculated (25).

At the final follow-up, pelvic function was evaluated using the Majeed scoring system (0–100 points). Scores were classified as excellent (≥85), good (70–84), fair (55–69), or poor (<55), with higher scores indicating better recovery (16). Perioperative parameters included operative time, intraoperative blood loss, and the number of fluoroscopic exposures recorded from skin incision to wound closure. Adverse events such as wound complications, neurovascular injury, implant failure, or secondary loss of reduction were documented.

### 2.7 Statistical analysis

Statistical analyses were performed using SPSS version 25.0 (IBM Corp., Armonk, NY, United States). Given the single-arm design and modest sample size, the analysis was primarily descriptive. Continuous variables are presented as median (interquartile range, IQR), and categorical variables are presented as counts and percentages. No formal hypothesis testing or between-group comparisons were planned.

## 3 Results

### 3.1 Patient characteristics and perioperative parameters

A total of 32 patients with unstable pelvic ring injuries were treated using the RAFR system during the study period. The cohort included 16 men and 16 women, with a mean age of 44 ± 16.7 years. Injuries comprised a spectrum of Tile type B and C patterns, and the majority of patients had associated injuries, including thoracic trauma, lumbar vertebral fractures, extremity fractures, and urogenital injuries (Table 1), reflecting the polytrauma context in which pelvic ring disruptions typically occur.

All procedures were completed with the planned minimally invasive, robot-assisted closed reduction and percutaneous fixation; no conversion to open reduction was required. The median operative time was 270 min (IQR 225–311), the median intraoperative blood loss was 150 mL (IQR: 100–300), and the median number of fluoroscopic exposures was 35 (IQR: 31–45). No intraoperative neurovascular injuries, iatrogenic fractures related to the robotic system, or device malfunctions were observed (Table 2).

**Table 2:** Clinical outcome of the patients treated for unstable pelvic fractures.

Case number	Operation time (min)	Blood loss (ml)	Fluoroscopic exposures	Residual displacement/mm	Evaluation	Majeed score
1	210	100	34	2	Excellent	Lost to follow-up
2	270	200	49	3	Excellent	70
3	200	200	31	4	Excellent	84
4	255	200	41	2	Excellent	72
5	360	100	32	3	Excellent	86
6	210	500	78	2.5	Excellent	77
7	240	50	26	3.5	Excellent	94
8	420	1,200	52	12.5	Fair	51
9	285	150	38	8	Good	66
10	230	150	26	2	Excellent	86
11	270	120	49	16	Fair	83
12	330	100	44	4	Excellent	92
13	360	100	37	3.8	Excellent	58
14	300	50	31	3	Excellent	91
15	510	1,500	59	9	Good	71
16	240	100	41	6	Good	89
17	240	100	37	9	Good	78
18	330	300	29	5	Good	Lost to follow-up
19	270	100	21	3.5	Excellent	90
20	390	300	22	3.5	Excellent	85
21	270	300	62	8	Good	78
22	210	250	34	8.5	Good	69
23	270	100	31	2.5	Excellent	77
24	270	20	26	1.5	Excellent	92
25	480	600	65	7.5	Good	56
26	120	50	25	5.5	Good	81
27	305	150	33	6.5	Good	74
28	290	100	34	5	Good	66
29	180	100	44	3.8	Excellent	58
30	240	500	54	3.6	Excellent	74
31	230	200	36	12.5	Fair	64
32	200	200	33	9	Good	90

### 3.2 Radiographic and functional outcomes

Postoperative CT measurements showed a median residual displacement of 4.0 mm (IQR: 3.0–8.0 mm). According to Matta’s criteria, reduction quality was excellent in 17 patients (53.1%), good in 12 (37.5%), fair in 3 (9.4%), and poor in none, yielding an excellent-to-good rate of 90.6%.

**Figure 3:**
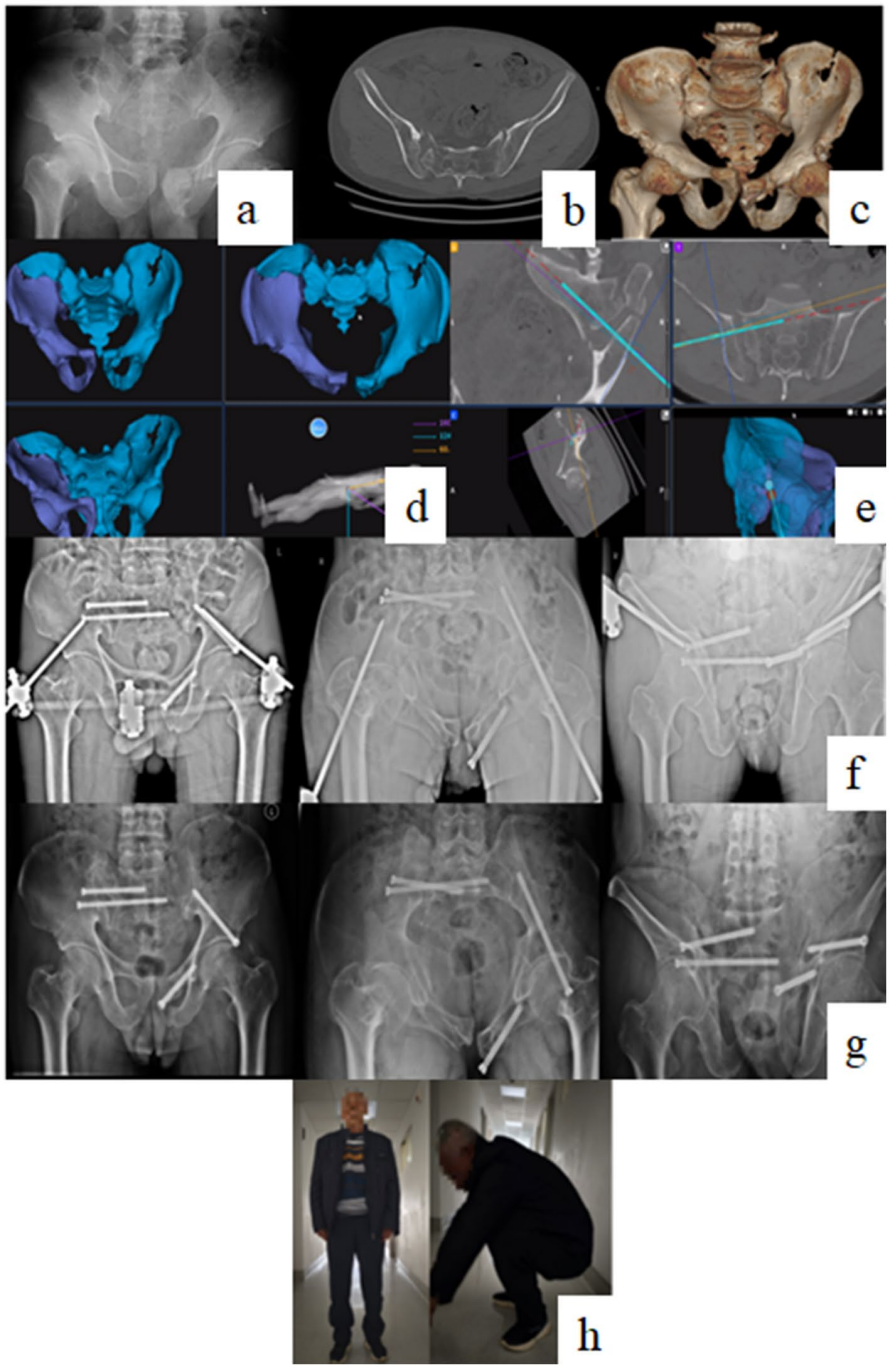
Representative clinical case of a Tile C pelvic fracture. **(a–c)** Preoperative radiographs, CT, and 3D reconstruction images; **(d)** Intraoperative reduction under navigational monitoring; **(e)** Guidewire placement for sacroiliac screw under navigation following reduction; **(f,g)** 1-month and 3-month postoperative follow-up radiographs; and **(h)** Lower limb functional status postoperatively.

Two patients were lost to follow-up after hospital discharge. The remaining 30 patients were followed for a mean of 12 weeks. At the final visit, no major complications such as deep infection, implant failure, or secondary loss of reduction were identified. The mean Majeed score was 76.7 ± 12.0 points, with the majority of patients achieving good or excellent functional categories (Figure 3).

## 4 Discussion

In this single-center case series, we describe the early clinical application and safety profile of an intelligent robot-assisted fracture reduction (RAFR) system for fresh, unstable pelvic ring injuries. To the best of our knowledge, this represents one of the first comprehensive reports on a dedicated pelvic reduction robot applied to a human cohort. In 32 consecutive patients with Tile B/C fractures, closed reduction was successfully completed in all cases without conversion to open reduction. The system achieved a median CT-based residual displacement of 4.0 mm and an excellent-to-good Matta rate of 90.6%. These findings suggest that robot-assisted closed reduction is not only clinically feasible but also capable of achieving radiographic alignment comparable to the upper range of outcomes historically reported for conventional closed techniques (5–9). Crucially, the safety profile was favorable; no neurovascular injuries, implant malpositions, or severe soft tissue complications were observed. This initial success validates the system’s core design philosophy: combining the precision of computer-assisted planning with the mechanical stability of robotic manipulation to standardize minimally invasive pelvic surgery.

The evolution of pelvic fracture management has long been defined by a challenging trade-off between anatomic restoration and physiological preservation. Open reduction and internal fixation (ORIF) offers direct visualization and superior reduction quality but constitutes a significant trauma to the patient. Conversely, conventional manual closed reduction has the advantage of minimizing trauma but is notoriously unpredictable. It relies heavily on 2D fluoroscopy, which provides limited spatial information and demands strenuous physical effort from the surgeon to counteract strong muscle contractions. This often results in suboptimal reduction or operator fatigue, ultimately forcing a conversion to open surgery. Our robotic workflow effectively resolves this clinical conflict. By enabling high-precision reduction through minimal incisions, the RAFR system aligns with damage control principles—preserving the soft tissue envelope and minimizing blood loss—while delivering the reduction quality typically associated with open surgery. This “best of both worlds” approach potentially transforms acute unstable fractures from chaotic, high-morbidity cases into manageable, programmed procedures.

A key differentiator of our system is its departure from the trial-and-error methodology of manual reduction (5, 7, 8). Traditional techniques remain heavily dependent on repeated fluoroscopic checks and manual manipulation, which leads to prolonged radiation exposure for the surgical team. In contrast, our workflow reconfigures the procedure around 3D imaging and computer-assisted planning. By mirroring the intact hemipelvis to create a patient-specific target, we eliminated the need for invasive fiducial markers and replaced subjective estimation with deterministic trajectory execution. Mechanically, the system’s functional separation between contralateral stabilization and ipsilateral reduction is a critical innovation. The contralateral hemipelvis is anchored via passive arms, providing a rigid mechanical reference that resists the forces applied during reduction. Clinically, this configuration is advantageous because the pins used for stabilization can often be incorporated into the definitive fixation (e.g., for INFIX constructs), thereby simplifying the construct and minimizing additional trauma (21, 23). Furthermore, unlike a human assistant who may fatigue, the robotic arm maintains the reduced position indefinitely. This “static hold” capability is vital; it allows the surgeon to insert guide wires and screws with precision, free from the urgency of losing the reduction, thereby significantly reducing the risk of screw malposition and neurovascular injury.

The versatility of the RAFR system is further highlighted by its application in non-standard patient populations. Our cohort included a 6-year-old patient with a severely displaced, unstable pelvic fracture following a traffic accident. Pediatric pelvic fractures present unique challenges: extensive open exposure is undesirable due to growth plate risks, and standard adult plates are often ill-suited to pediatric anatomy. Using the robotic system, we achieved anatomic reduction and performed percutaneous K-wire fixation. The robot’s ability to execute fine, scale-adjusted maneuvers allowed for a fixation strategy that was minimally invasive yet stable enough to permit early mobilization. Follow-up revealed excellent functional recovery. This case suggests that the system’s benefits—precision planning and soft tissue preservation—may be particularly amplified in pediatric trauma or other scenarios where standard implants and exposures are contraindicated.

While the system automates the reduction maneuver, safe implementation requires a rigorous “surgeon-in-the-loop” workflow. Our early experience revealed several technical nuances critical for success. First, image quality is paramount. Preoperative protocols must include bowel preparation to minimize intestinal gas, which can obscure bony landmarks on intraoperative fluoroscopy and CBCT. Additionally, close coordination with the anesthesia team is essential during the registration scan; controlling respiratory excursion ensures that the optical tracking of the anterior superior iliac spine (ASIS) remains accurate. Second, tracker stability is the Achilles’ heel of any navigation system. We encountered one instance of tracking drift due to loosening of the reference frame, necessitating re-registration. Rigorous fixation of trackers to the bone cortex is non-negotiable. Third, force management requires a hybrid approach. The robotic arm is safety-limited to a maximum force. In cases of fracture impaction or interlocking fragments, this limit may be exceeded, causing the robot to pause. We found that a collaborative strategy is effective: the surgeon performs a manual “unlocking” maneuver to disengage the fragments, after which the robot resumes its trajectory to complete the fine reduction. This synergy ensures that the robot does not blindly force a reduction against osseous resistance, acting as an essential haptic safety layer. Finally, contingency planning remains vital. Despite the high success rate, the complexity of the integrated software-hardware architecture means that technical faults are possible. A strictly defined protocol for converting to manual or open reduction must be in place, ensuring patient safety is never compromised by equipment downtime.

## 5 Limitations

This study has several important limitations. First, it is a retrospective single-center case series with a relatively small sample size and no concurrent control group treated with conventional manual or open reduction. Therefore, the findings should be interpreted as preliminary evidence of feasibility and short-term safety rather than as proof of superiority over existing techniques. Second, the follow-up duration was limited to approximately 3 months, precluding assessment of long-term outcomes such as post-traumatic arthrosis, implant longevity, or late functional decline. Third, the current RAFR workflow requires an intact or minimally displaced contralateral hemipelvis as a template, and patients with bilateral severe comminution or deformity were excluded; the results therefore may not be generalizable to the most complex bilateral patterns. Future multicenter comparative studies, ideally randomized where feasible, are needed to confirm these early findings, define the specific fracture patterns and patient subsets most likely to benefit from robot-assisted closed reduction, and evaluate cost-effectiveness and learning-curve effects.

## 6 Conclusion

This case series reveals that an intelligent robot-assisted fracture reduction system can be safely integrated into the minimally invasive management of fresh, unstable pelvic ring injuries, achieving high rates of satisfactory CT-based reduction and encouraging early functional outcomes without conversion to open surgery. While these results support the feasibility of this approach, they should be considered as a foundation for further research. Larger multicenter studies with control groups and longer follow-up are required to clarify the comparative effectiveness, indications, and implementation strategies for robot-assisted closed reduction in pelvic ring trauma.

## Data Availability

The original contributions presented in the study are included in the article/supplementary material, further inquiries can be directed to the corresponding author.
